# A Rapid Method for Detecting Microplastics Based on Fluorescence Lifetime Imaging Technology (FLIM)

**DOI:** 10.3390/toxics10030118

**Published:** 2022-03-02

**Authors:** Fang Zhou, Xin Wang, Guangxin Wang, Yanxia Zuo

**Affiliations:** The Analysis and Testing Center, Institute of Hydrobiology, Chinese Academy of Sciences, Wuhan 430072, China; zhoufang@ihb.ac.cn (F.Z.); wangxin@ihb.ac.cn (X.W.); wanggx@ihb.ac.cn (G.W.)

**Keywords:** microplastics, fluorescence lifetime, phasor analysis, fingerprint library

## Abstract

With the increasing use and release of plastic products, microplastics have rapidly accumulated in ecological environments. When microplastics enter the food chain, they cause serious harm to organisms and humans. Microplastics pollution has become a growing concern worldwide; however, there is still no standardized method for rapidly and accurately detecting microplastics. In this work, we used fluorescence lifetime imaging technology to detect four kinds of Nile red-stained and unstained microplastics, and the unique phasor fingerprints of different microplastics were obtained by phasor analysis. Tracing the corresponding pixels of the “fingerprint” in the fluorescence lifetime image allowed for the quick and intuitive identification of different microplastics and their location distributions in a mixed sample. In our work, compared with staining the four microplastics with a fluorescent dye, using the phasor “fingerprint library” formed by the autofluorescence lifetimes of the microplastics was more easily distinguished than microplastics in the mixed samples. The feasibility of this method was further tested by adding three single substances—SiO_2_, chitin and decabromodiphenyl ethane (DBDPE), and surface sediments to simulate interferent in the environment, and the results providing potential applications for the identification and analysis of microplastics in complex environments.

## 1. Introduction

Thompson [1] first proposed the scientific term “microplastics” in 2004. Microplastics are any synthetic solid particle or polymeric matrix, with regular or irregular shape and with size ranging from 1 μm to 5 mm, of either primary or secondary manufacturing origin, which are insoluble in water [2]. The structures and properties of microplastics are extremely stable, the degradation process is very slow, and microplastics can exist in the environment for a long time [3]. Because of their small size, microplastics are easily eaten by fish and other aquatic animals in the water, and remain in organisms for a long time [4,5], posing a serious threat to fish reproduction [6,7,8]. In addition, because of their large specific surface area, microplastics can act as carriers and can easily interact with toxic compounds such as heavy metals or hold organic pollutants to form dangerous pollutants [9,10]. Once these new pollutant combinations are ingested by aquatic organisms, they accumulate in different tissues and organs and are magnified through the food chain, resulting in serious consequences for aquatic organisms and even humans [11].

Microplastics differ from other components in the environmental matrix in terms of shape, color, and composition, and there are differences among microplastics from different sources. Therefore, the qualitative and quantitative analysis of microplastics is difficult. To date, there is no completely unified testing standard [12,13,14]. The current methods for identification and quantitative analysis of microplastics include visual inspection, Fourier transform–infrared spectroscopy, Raman spectroscopy, and pyrolysis/gas chromatography–mass spectrometry. The visual inspection method selects and classifies plastics according to color and shape by visual observation or with the assistance of a microscope. This method is simple to operate, low cost, and nontoxic, and is suitable for preliminary research and screening of samples. However, it can only be performed on larger microplastics, such as the size of particles visible to the naked eye (greater than ~500 μm). Plastics are manually separated by size, color, and type (fragments, pellets, or beads) and then counted [15]. Errors in judgment easily occur due to the subjective factors of the operator and the influence of environmental media [16,17,18]. Even for the most experienced analysts, it is challenging to distinguish between microplastics and other organic or inorganic fragments, such as quartz particles, plant chunks, or animal parts [19]. 

Fluorescent staining of microplastics with Nile red is a promising visual method, permitting fast and easy recognition of plastic particles [20], Results can be seen under orange, red, or green filters in a fluorescence microscope [21]. When compared with other dyes, Nile red has the advantages of high adsorption to plastics and good affinity for various polymers [22,23,24,25]. It effectively dyes the surface of microplastics and represents a novel method for tracking microplastics with fluorescent markers, providing a rapid means for evaluating the abundance of microplastics [26,27]. However, the staining effect depends on factors such as the dye concentration, solvent type, and temperature, and the subsequent changes in fluorescence intensity may affect the repeatability of detection [28]. Uncontrollable technical deviations are introduced during the plastic measurement process itself or during environmental testing of the sample [29]. 

Vibrational spectroscopy methods are nondestructible and complementary, producing a spectrum based on the interaction of light with molecules: FTIR produces infrared spectrum based on changes in dipole moments, while Raman spectroscopy obtains inelastic scattered light of different frequencies through molecular vibration, thereby obtaining the fingerprint of the chemical structure of the material [30]. By comparing the spectral results with those of known plastic polymers, the polymer types of microplastics can be identified quickly and directly [31]. Fourier transform infrared spectroscopy (micro-FTIR) is to connect a microscope to a Fourier transform infrared spectrometer, and this method can meet the detection requirements of microplastics with a particle size larger than 10 µm [32]. However, during the detection process, only one microplastic particle picked out from the sample can be analyzed at a time, which makes the operation process cumbersome. FTIR cannot be performed in the presence of water in the sample as it may overlay the target spectrum [33]. 

Raman spectroscopy has higher spatial resolution and is insensitive to sample thickness, shape, and interference signals from H_2_O and CO_2_ [34]. However, the inspection of small filter areas makes the entire process labor-intensive and time-consuming. Another noteworthy problem is spectral interference from matrices. For example, different additives, pigments, and compounds in bottled mineral water (such as calcium chloride, magnesium fluoride, and silicon dioxide), which produce stronger background signals that counteract a weak Raman signal [35]. 

Pyrolysis/gas chromatography–mass spectrometry (Pyr/GC–MS) is an analytical technique that thermally degrades a sample into small molecular fragments by pyrolysis, which is then separated and identified using GC–MS [36]. Pyr/GC–MS can accurately identify different polymer types, and it also provides information on additives in microplastics [37]. Additionally, the detection is in the millimeter range, thus giving low efficiencies and resolutions [34]. However, the microplastics must be placed into the pyrolysis tube, and it takes about 70 min to run the analysis of one particle at a time [19]. It is also destructive to the sample, which leads to complete fragmentation of the particles, thus affecting further particle analysis [38]. The advantages and limitations of these methods coexist, so the joint use of multiple methods and the development of new methods that complement existing methods are the ways to solve the problem.

Fluorescence lifetime imaging microscopy (FLIM), which is used in this study, uses a femtosecond pulsed laser with a high repetition rate to excite fluorescence from the sample. The fluorescence decay that occurs after the excitation pulse is directly recorded with time-correlated single photon counting (TCSPC), and the variations in fluorescence intensity (or number of photons) with time are determined. The fluorescence lifetime of the material is obtained by curve fitting [39]. The fluorescence lifetime reflects the rate at which a fluorescent molecule de-excites from the excited state to the ground state, which is an inherent characteristic of fluorescent molecules, with the changes in the fluorescence lifetime sensitively reflecting changes in the microenvironments of the fluorescent molecule [40]. Measurements of fluorescence lifetime are not affected by the fluorescence probe concentration, excitation light intensity, photobleaching, or other factors, so they have the advantages of strong specificity, high sensitivity, and the ability to perform quantitative measurements [41,42]. In recent years, FLIM technology has enabled important research progress in practical applications for biomedicine, material science, and many other fields [43,44,45,46]. FLIM technology is often used to detect the distribution of cell microenvironmental parameters and the state of energy metabolism, as well as to characterize the internal structure and properties of materials [47]. The processing of fluorescence lifetime data is also a critical component of the process. Phasor analysis is a visual fluorescence lifetime analytical method that visualizes fluorescence decay data without fitting [29], and it is much faster than traditional fitting methods. By converting the data to Fourier space, it is possible to understand the fundamentals of very complex processes such as dye photophysics, Förster resonance energy transfer (FRET), tissue autofluorescence anatomical pathology, and cellular dynamics [48]. Therefore, in recent years, phasor analysis has shown great advantages in quantitative analyses, visualization, and cluster analyses of fluorescence lifetime data.

Monteleone et al. [49] discovered the potential of autofluorescence of plastics while working on a plastic particle sample extraction method. Autofluorescence refers to the light emitted in the ultraviolet–visible and near-infrared spectrum when a substance is excited by light of the appropriate wavelength [50]. It is an inherent, naturally occurring fluorescence signal that does not require labeling. In recent years, the application of autofluorescence has mainly been in the fields of medicine and plant research [51,52], and little research has been done in the field of substances such as microplastics. Recently, two studies have used fluorescence lifetime imaging technology to identify Nile red stained and unstained microplastics [53,54], respectively, which proved the feasibility of this technique to characterize the morphology and fluorescence lifetime of microplastics. However, no research has confirmed that this technique can effectively detect and analyze microplastics in the presence of matrix interferents or even complex environmental matrices.

In this study, the autofluorescence spectra of microplastics were analyzed using the continuous excitation lines of a pulsed laser to determine the detection range of microplastics. This technique was used to detect stained and unstained microplastics, and the phasor analysis parameters were manually calibrated, the results demonstrate the advantage of using the fluorescence lifetime fingerprint of microplastic autofluorescence to differentiate mixed microplastics. To further test the feasibility of the method in the presence of matrix interference, a variety of interferences were added, and different microplastics could still be accurately identified through the use of several microplastic fingerprint libraries established in this study.

## 2. Materials and Methods

### 2.1. Preparation of Microplastic Samples

In this study, four kinds of microplastic particles with sizes of approximately 100 µm were used: acrylonitrile butadiene-styrene copolymer (ABS), polyethylene terephthalate (PET), polyvinyl chloride (PVC), and poly (lactide) (PLA), and were purchased from Guangzhou Science and Chemical Company, China. ABS, PET, and PLA were obtained by crushing at low temperature, and PVC was formed by direct polymerization. A Nile red staining solution was prepared with a final concentration of 0.5 μM in acetone. The Nile red staining solution was added to the four microplastics, with a microplastic concentration of 50 mg/mL, and they were incubated in a constant temperature incubator at 70 °C for 1 h; 100 μL of each stained sample was mixed to obtain a mixed sample containing four kinds of microplastics.

### 2.2. Laser Scanning Confocal Microscopy and Spectral Analysis

A laser scanning confocal microscope (Leica TCS SP8) was used to collect fluorescence and bright field images. An HC PL APO 20X/0.75 dry objective lens was used for collection. The autofluorescence of microplastics was excited by a continuous wave 405 nm laser and a 440 nm pulsed white light laser (WLL), and fluorescence and bright field images were collected. An xyλ scan was used to detect the autofluorescence emission spectra of the microplastics from 415–850 nm and 455–850 nm with the 405 nm laser and a 440 nm laser, with a step size of 20 nm. The xyΛλ scanning was performed to collect spectral images at different excitation wavelengths (WLL, 440–550 nm range, step size 10 nm) and emission wavelength detection ranges (415–600 nm, step size 10 nm, detection window 15 µm), and Leica spectral analysis software was used to excite and detect fluorescence.

### 2.3. Fluorescence Lifetime Imaging Microscopic Detection

Fluorescence lifetime imaging of microplastic particles was carried out by using a Leica TCS SP8-Falcon (Rapid Life Imaging) system. A pulsed WLL was used to excite the microplastics, and a HyD X detector was used for photon detection. In this study, Nile red-stained microplastics were excited by 500 nm WLL and detected in the range 520–570 nm. Unstained microplastics were excited by 440 nm WLL to detect signals in the range 455–545 nm, and the detection parameters were determined by the results of spectral analysis. Frame accumulation was set to 50 and 100 to obtain a sufficient number of photons. Images measuring 512 × 512 pixels were collected at 400 Hz scanning frequency. The pulse repetition rate was 80 MHz.

### 2.4. Data Analysis

**Figure 1:**
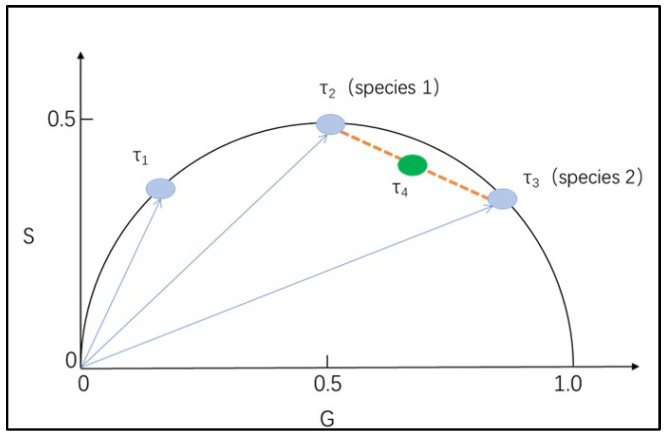
Schematic diagram of phasor transformation; τ_1_, τ_2_, and τ_3_ are located on the universal circle. The shift of the phasor along the universal circle with changes in the single exponential lifetime occur in the order τ_1_ > τ_2_ > τ_3_. The phasor distribution of a mixture of two species (species 1 and species 2) falls on the line connecting the two components, and its position can be determined by the relative fractional contributions.

**Figure 2:**
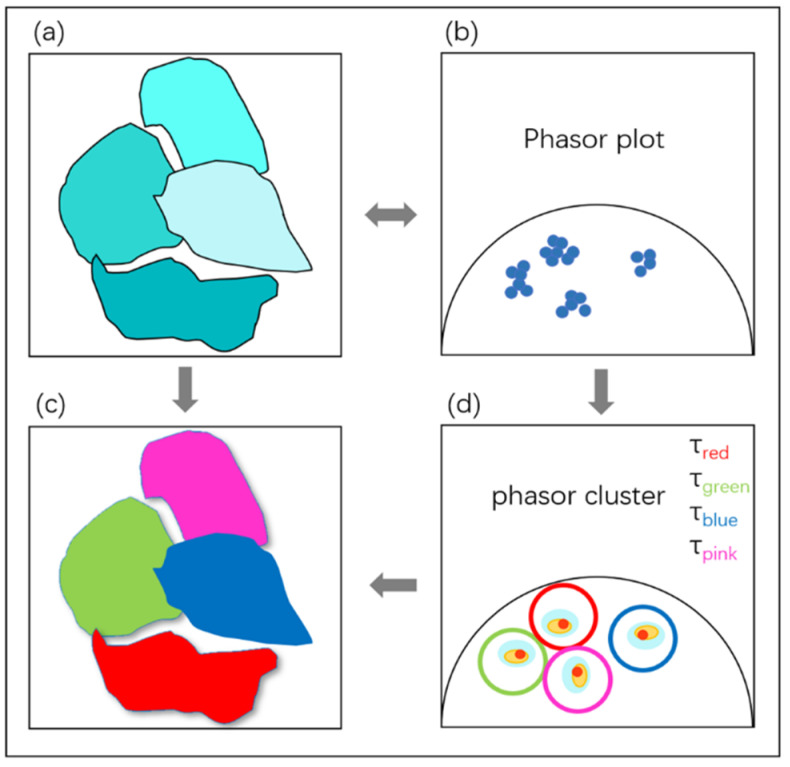
Schematic diagram of the Phasor-FLIM phasor analysis flow: (**a**) fluorescence intensity map containing unprocessed lifetime information; (**b**) lifetime phasor map obtained by FLIM-phasor analysis; (**c**) circle selection and acquisition of lifetime phasor clusters and the corresponding fluorescence lifetime values; (**d**) fluorescence lifetime maps of different phasor clusters obtained by phasor cluster analysis and pseudocolor labeling.

The fluorescence lifetimes of the images were analyzed by using Phasor-FLIM phasor analysis software. The concept of the universal circle in phasor images was defined by Jameson [55]. The universal circle is a semicircle with a radius of 0.5 that centered at (0.5, 0.0). As shown in Figure 1, each pixel in the fluorescence lifetime measurement map can be mapped to the corresponding phasor point in the phasor diagram, which is represented by coordinates (G, S). Each point on the semicircle represents a different lifetime, with the lifetime value decreasing from left to right. Longer lifetimes occur near the coordinates (0, 1), while shorter lifetimes occur near (0, 0) [48]. The phasor points resulting from the transformation of fluorescence lifetime information, whose positions depend on the number of photons each responsible fluorophore contributes to the phasor calculation. Therefore, phasors for single-exponential species are located in the universal circle. The phasor of a system composed of two different pure single-exponential species falls on the straight line that connects the phasor locations of the individual species in the semicircle [56,57,58]. Therefore, the phasor of a multicomponent sample is located inside the universal circle. Figure 2 shows a schematic diagram of the phasor transformation process. During phasor analysis, the fluorescence decay information from each pixel in the FLIM image is transformed into a point in the phasor plot. The phasor points corresponding to pixels with similar fluorescence decay characteristics are displayed in similar positions; this process forms a certain cluster distribution, known as a “phasor cluster”. This transformation allows for the analysis of different lifetime phasor clusters that can then be mapped back onto the image to create the FLIM images [59]. By circling the phasor clusters of interest with the cursor in the phasor diagram and finding the positions of the pixels corresponding to the phasor points on the sample through corresponding relationships, the regions with similar fluorescence characteristics in the sample can be marked, and vice versa. When different phasor cluster circles are selected with different pseudocolors, this method can also be used to realize a polychromatic display of fluorescence lifetime images [29].

### 2.5. Verification Experiments with Single Impurities and Complex Sediments

SiO_2_(CAS: 60676-86-0) and chitin (CAS: 1398-61-4) were purchased from Shanghai Macklin Biochemical Co., Ltd. (Shanghai, China). Decabromodiphenyl ethane (DBDPE, CAS: 84852-53-9) was purchased from Yantian Biotechnology Co., Ltd. (Shanghai, China). The same quantities of the three interfering substances were mixed with the four kinds of microplastics, and then the feasibility of the method was verified by FLIM.

Surface sediments were provided by the Purification and Restoration Ecology Laboratory of the Institute of Aquatic Biology, Chinese Academy of Sciences [60,61]. Surface sediment of West Lake was collected by a Peterson dredge with three replicates; the three samples were then mixed to create one sample. The sediment samples were stored in air-sealed plastic bags at 4 °C before laboratory testing. The sediment was diluted in suspension to 100 mg/mL, and 10 μL of sediment dilution was mixed with a mixture of ABS, PET, PVC, and PLA to verify the effect of the method.

### 2.6. Verification Experiment with Raman Spectroscopy

In order to compare the proposed analytical technique with the widely used instrumentation, microplastics were identified using a Renishaw inVia Raman microscope (Wotton-under-Edge, Gloucestershire, UK). A 785 nm laser was used for Raman analysis, and the Raman spectra in the 300–3200 band were recorded to identify the composition of the compounds [62]. The Raman spectra of the samples were compared with the Raman spectral library and the spectra of standard polymers to verify the polymer types of four kinds of microplastics. Microplastics and surface sediments were mixed (as described in Section 2.6), and the detection effects of Raman spectroscopy were compared.

## 3. Results

### 3.1. Bright Field Image Characteristics of Microplastics

**Figure 3:**
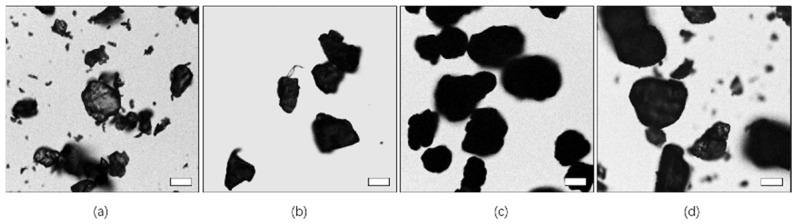
Representative optical images of microplastics: (**a**–**d**) acrylonitrile-butadiene-styrene copolymer (ABS), polyethylene terephthalate (PET), polyvinyl chloride (PVC), and poly(lactide) (PLA), respectively. The scale bar indicates 50 µm.

The four kinds of microplastics were imaged by optical microscopy (Figure 3). The four microplastics were approximately 100 µm in size after sieving and mixing with a small amount of smaller microplastics. ABS, PET, PVC, and PLA were all granules and could not be clearly distinguished by the naked eye.

### 3.2. Measurement of Excitation and Emission Spectra of Microplastics

**Figure 4:**
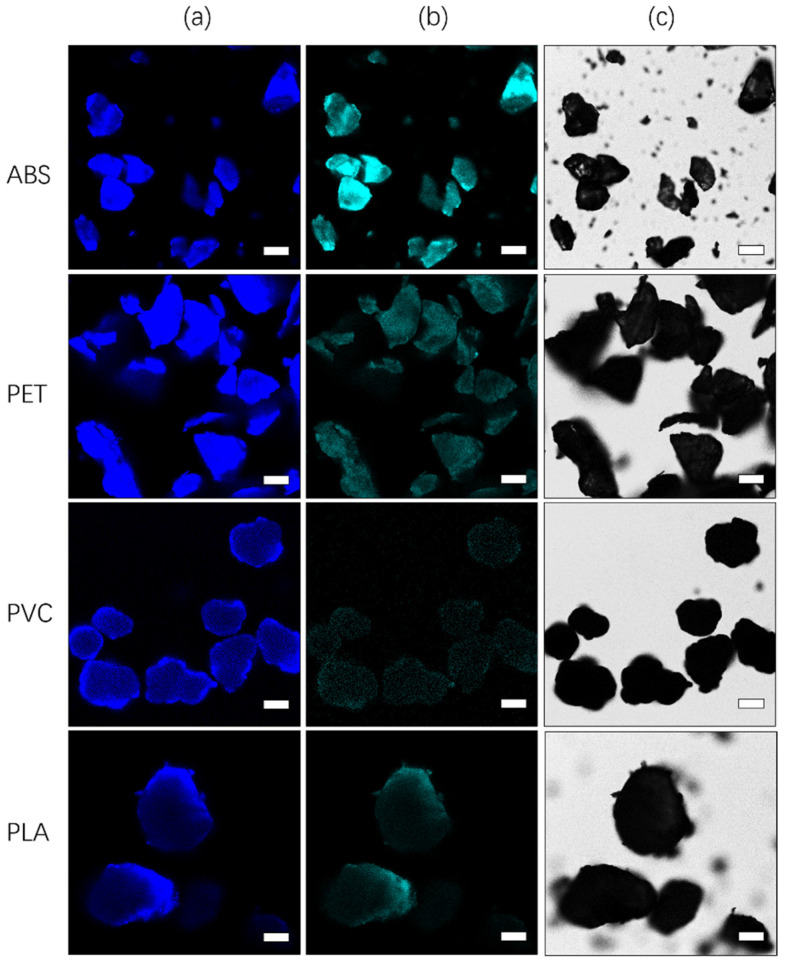
Fluorescence images resulting from excitation with different lasers and bright field images. (**a**) Autofluorescence images of ABS, PET, PVC, and PLA excited with a 405 nm laser; (**b**) autofluorescence images of ABS, PET, PVC, and PLA excited with a 440 nm laser; (**c**) white light images of ABS, PET, PVC, and PLA. The scale bar indicates 50 µm.

Images were collected under the same experimental conditions, and the autofluorescence of microplastics was excited with 405 and 440 nm lasers. The results shown in Figure 4 indicate that for the autofluorescence of microplastics, short-wavelength excitation with a 405 nm laser in the ultraviolet band is more effective, and it results in stronger absorption and a higher photon conversion rate.

**Figure 5:**
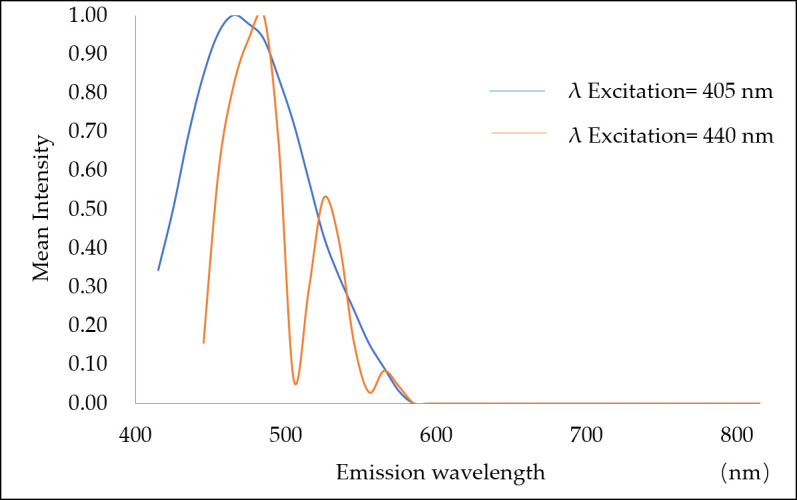
Autofluorescence emission spectra of ABS excited by 405 and 440 nm lasers.

The emission spectra of the four kinds of plastics were detected by confocal microscopy in xyλ mode, and they were basically identical. The ABS data are shown as an example in Figure 5. When excited with the 405 nm laser, the maximum emission peak was 465 nm. When excited with the 440 nm laser, there were three emission peaks at 485, 525, and 565 nm. The emission spectra were all concentrated at wavelengths below 600 nm.

**Figure 6:**
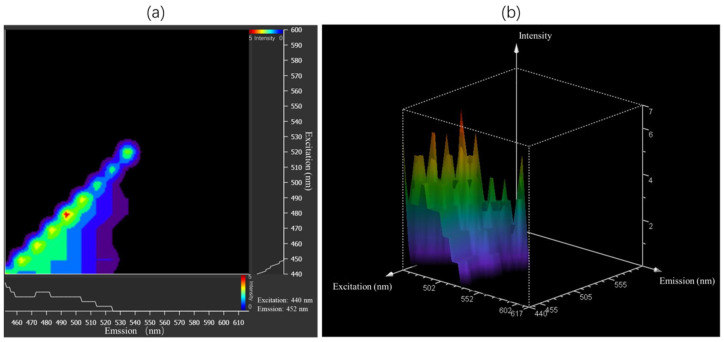
Excitation and emission spectral scans of microplastics. Taking ABS as an example, the horizontal axis is the emission spectrum detection range and the vertical axis is the excitation spectrum range. (**a**) ABS excitation/emission contour plot; (**b**) ABS excitation/emission 3D view.

Furthermore, the wavelength range of the WLL pulsed laser was effective in exciting autofluorescence from the microplastics. For each sample, a step size of 10 nm was used over the range 440–600 nm. Taking the 10 µm detection range as the emission bandwidth, the fluorescence emission spectrum was obtained over the range 445–620 nm. Taking as an example the excitation and emission spectra of ABS determined with xyΛλ scanning, the results in Figure 6 show that autofluorescence from microplastics can be excited within the range 440–520 nm, and the fluorescence emission spectrum is mainly within the range 445–545 nm. There was no fundamental difference in the photon conversion rates resulting from excitation within the range 440–520 nm. Therefore, 440 nm was the shortest wavelength and gave the best penetration depth for the microscope we used. Subsequent experiments used 440 nm WLL excitation and 445–545 nm emission bandwidth detection.

### 3.3. Fluorescence Lifetime Measurements and Phasor Analytical Procedures for Nile Red-Stained and Unstained Microplastics

**Figure 7:**
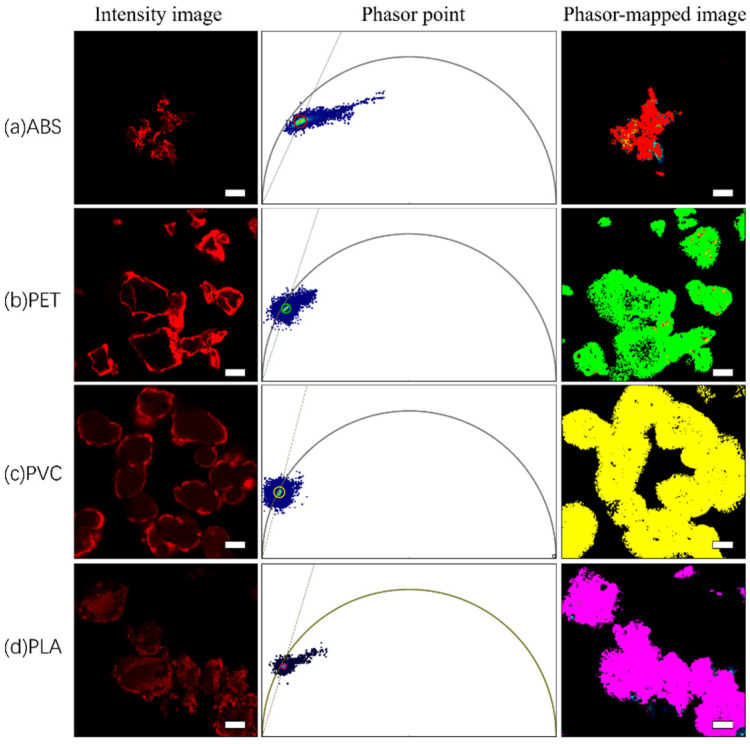
Application of phasor analysis to Nile red-stained microplastics: (**a**) fluorescence intensity image, phasor map, and fluorescence lifetime map for phasor cluster area mapping of Nile red-stained ABS, which corresponds to a fluorescence lifetime τ ≈ 2.2 ns; (**b**) fluorescence intensity image, phasor map, and fluorescence lifetime map for phasor cluster area mapping of Nile red-stained PET, which corresponds to a fluorescence lifetime τ ≈ 3.2 ns; (**c**) fluorescence intensity image, phasor map, and fluorescence lifetime map for phasor cluster area mapping of Nile red-stained PVC, which corresponds to a fluorescence lifetime τ ≈ 3.8 ns; (**d**) fluorescence intensity image, phasor map, and fluorescence lifetime map for phasor cluster area mapping of Nile red-stained PLA, which corresponds to a fluorescence lifetime τ ≈ 3.3 ns. The scale bar indicates 50 µm.

**Figure 8:**
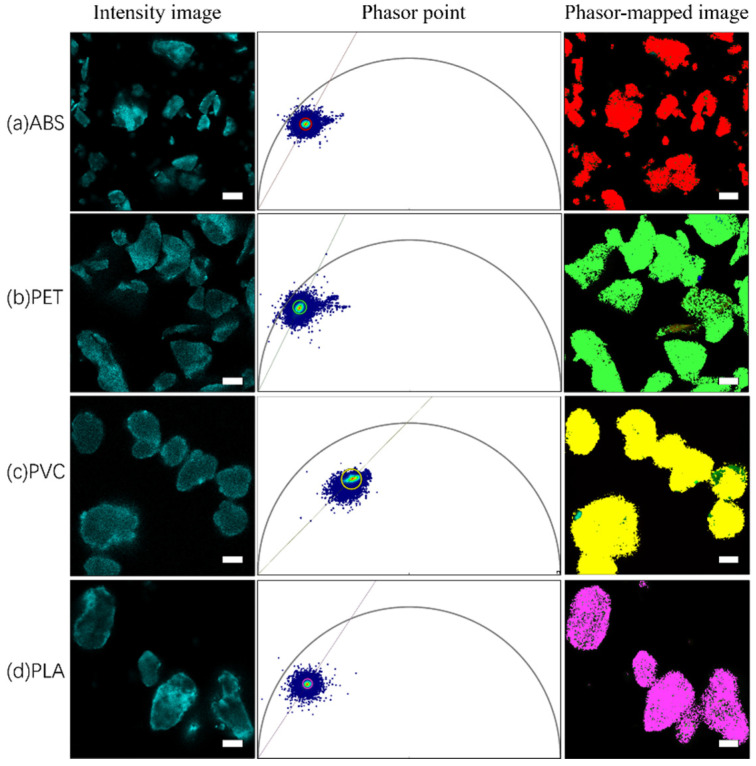
Application of phasor analysis to unstained microplastics: (**a**) fluorescence intensity image, phasor map, and fluorescence lifetime map for phasor cluster area mapping of unstained ABS, which corresponds to fluorescence lifetime τ ≈ 2.0 ns; (**b**) fluorescence intensity image, phasor map, and fluorescence lifetime map for phasor cluster area mapping of unstained PET, which corresponds to fluorescence lifetime τ ≈ 2.2 ns; (**c**) fluorescence intensity image, phasor map, and fluorescence lifetime map for phasor cluster area mapping of unstained PVC, which corresponds to a fluorescence lifetime τ ≈ 1.6 ns; (**d**) fluorescence intensity image, phasor map, and fluorescence lifetime map for phasor cluster area mapping of unstained PLA, which corresponds to a fluorescence lifetime τ ≈ 1.1 ns. The scale bar indicates 50 µm.

The fluorescence intensities and fluorescence lifetimes of Nile red-stained and unstained ABS, PET, PVC, and PLA microplastics were measured. Figure 7 and Figure 8 show the phasor images and corresponding fluorescence lifetime images of Nile red-stained and unstained microplastics, respectively, as well as the fast fluorescence lifetime τ values displayed by the software in real time; the τ values corresponds to phasor cluster positions in the universal circle.

Phasor analysis, which graphically depicts the distribution of fluorescence lifetimes, makes data interpretation fast and simple, especially for substances with complex photosystems such as microplastics. The corresponding phasor clusters in the phasor map were obtained by using colored circles, and then the corresponding pixels represented by the selected lifetime were mapped to the fluorescence lifetime image with the same pseudocolors. The pseudocolors were assigned as follows: red (ABS), green (PET), blue (PVC), and pink (PLA). The fluorescence lifetimes of each Nile red-stained and unstained microplastic is reflected at a specific position in the phasor map by phasor analysis, and this position can be considered the specific phasor fingerprint of the microplastic.

### 3.4. Phasor “Fingerprint Library” of Microplastics

A specific phasor cluster in each phasor image constitutes a phasor fingerprint, and multiple phasor fingerprints are gathered in a phasor image to form a phasor “fingerprint library”, which is similar to the comparison database of FTIR and Raman spectroscopy. Each kind of microplastic in the “fingerprint library” produces a specific phasor diagram, which contains phasor cluster location and rapid fluorescence lifetime information, as a reference database for subsequent identification of microplastics. Whether all kinds of microplastics can be identified quickly and easily in the “fingerprint database” depends on whether phasor fingerprints can be distinguished within the space in the picture. To optimize the phasor analysis protocol, the phasor calibration parameters were manually modified to make them more suitable for the phasor analysis in this study. The calibration parameters included the harmonic, threshold, and filters. “Harmonic” represents the corresponding lifetime multiplied by the harmonic order of the Fourier transform; an optimal value of 2 was selected in this study to shift the visualization of the phasor cluster region to a larger phase angle in the center for easy distinction. “Threshold” was set to 100 to filter out stray phasor point artifacts of microplastics from the phasor map. The “Median filter” was selected and set to 5, which smoothed the image to better highlight and enhance the visibility of details in the image. The optimized phasor calibration parameters were used to obtain the data for unified analysis in this study.

**Figure 9:**
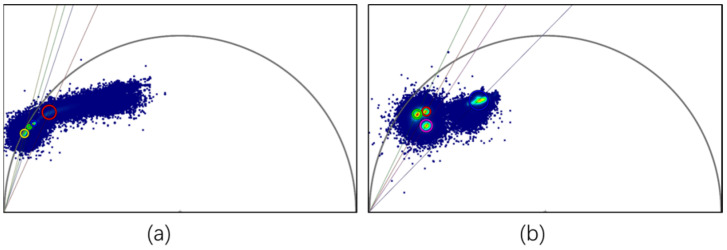
Phasor “fingerprint database”: (**a**) synchronous phasor analysis diagram for four microplastics stained with Nile red and (**b**) synchronous phasor analysis diagram for four unstained microplastics.

In Figure 9, the phasor images for the four Nile red-stained and unstained microplastics were assembled in a single phasor image to form a phasor “fingerprint library”. Nile red emission decays with single-exponential decay (τ = 3.1 ns) [54], which should be located on the omnipotent circle. After dyeing the different microplastics, Nile red emission changed from single-exponential decay to multiexponential decay when combined with microplastics. The results shown in Figure 5 indicate that the fluorescence lifetimes of Nile red-stained microplastics shifted to the interior of the universal circle in the corresponding phasor image. However, it is not easy to distinguish these different “fingerprints” by spatial distribution, which indicates that Nile red components still play an important role in fingerprint formation, and the corresponding phasor clusters are concentrated near the universal circle. In addition, since the staining of microplastics with Nile red may only remain at the physical coverage level or be affected by solvent polarity, the ratio and manner of dye–microplastic binding may affect the fluorescence lifetime results, thereby affecting the spatial distribution in the phasor diagram image. Therefore, it is necessary to further explore suitable combinations of Nile red and microplastics to establish a phasor “fingerprint library” that is readily distinguishable. In this case, the four undyed microplastics exhibit obvious spatial distances in the phasor “fingerprint database”, it is easy to distinguish them intuitively; there is no influence from external components, and the fluorescence lifetime results are stable and reliable.

### 3.5. Identification and Differentiation of Microplastic Mixtures

**Figure 10:**
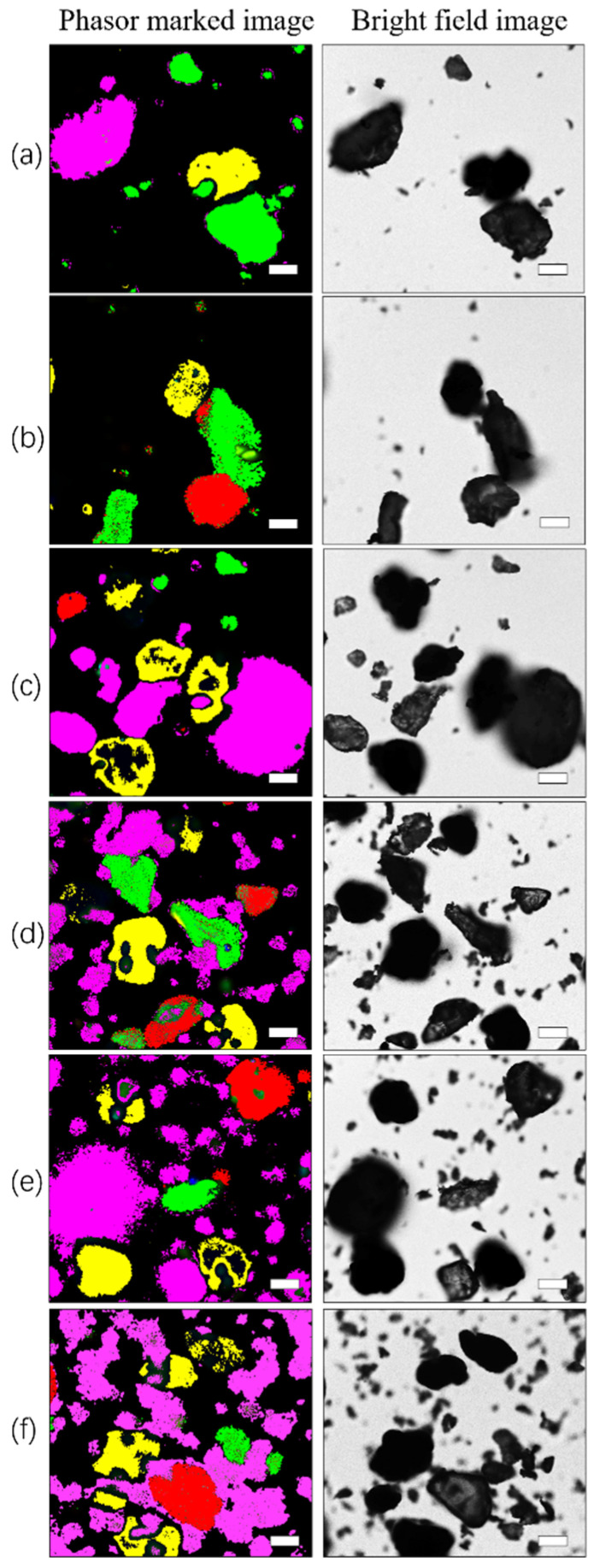
Identification and distinction of different microplastics: (**a**–**f**) phasor marked images on the left and bright field images on the right. The scale bar indicates 50 µm.

After verification of these experiments, and based on the phasor analysis process, the unstained microplastic phasor “fingerprint database” could be used to identify and distinguish mixtures. A single fluorescence lifetime map of the microplastic mixture was collected, and each phasor cluster in the phasor map was indicated with circles of different colors. The phasor clusters that were not obvious were selected at the corresponding position of the universal circle with known microplastic fluorescence lifetime τ. The corresponding pixels mapped in the fluorescence lifetime map were assigned different pseudocolors. Figure 10 shows an application of the microplastic phasor “fingerprint database”. By using the circles of different phasor clusters, different microplastics can be distinguished quickly and easily in the image, and the phasor diagram for the same spatial distribution can be found in the phasor “fingerprint database”. The microplastics in the mixed sample can thus be determined. In the identification results, ABS, PET, PVC, and PLA microplastics were marked with red, green, yellow, and pink, respectively, and the sizes and morphological characteristics of the identified microplastics could be recorded. The variety of pseudocolor-marked areas also clearly showed the distribution of each component. PET, PVC, and PLA are distributed in Figure 10a. ABS, PET, and PVC are distributed in Figure 10b. ABS, PET, PVC, and PLA are shown in Figure 10c–f.

### 3.6. Detection of Microplastics in Single Impurities and Complex Sediments

**Figure 11:**
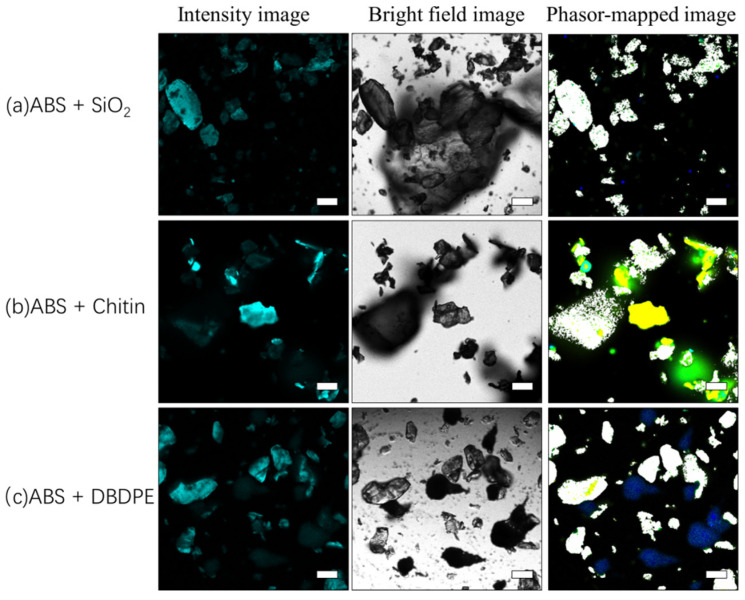
Identification of microplastics in a single interfering substance. (**a**) Fluorescence intensity image, bright field image, and fluorescence lifetime map of the phasor cluster area map of the mixture of ABS and SiO_2_; (**b**) fluorescence intensity image, bright field image, and fluorescence lifetime map of the phasor cluster area map of the mixture of ABS and chitin; (**c**) fluorescence intensity image, bright field image, and fluorescence lifetime map of the phasor cluster area map of the mixture of ABS and DBDPE. ABS was marked with white. The scale bar indicates 50 µm.

**Figure 12:**
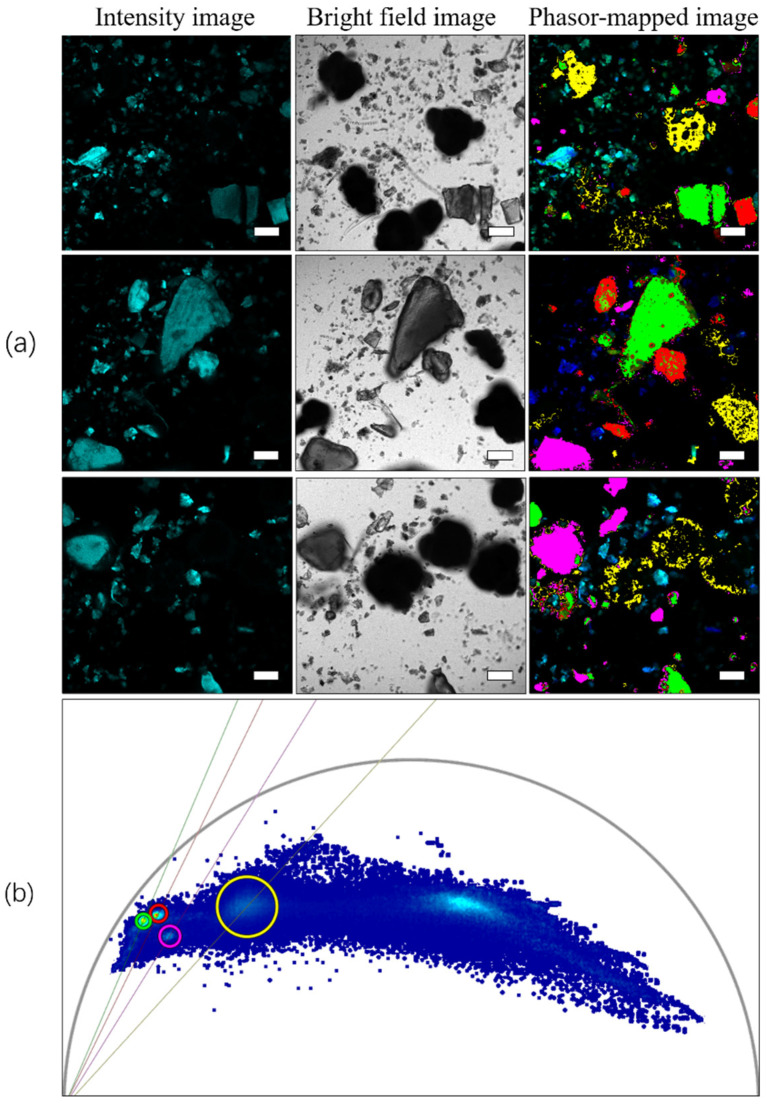
Identification of microplastics in sediments. (**a**) Fluorescence intensity image, brightfield image, and fluorescence lifetime map of phasor cluster area mapping of the mixture of four microplastics and sediments; (**b**) synchronous phasor analysis diagram for four microplastics and sediments. ABS, PET, PVC, and PLA microplastics were marked with red, green, yellow, and pink, respectively. The scale bar indicates 50 µm.

In the verification experiment involving the mixing of four microplastics with a single interferent, whether SiO_2_, chitin, or DBDPE, fluorescence lifetime imaging could effectively distinguish and identify microplastics. As shown in Figure 11, the phasor cluster position and lifetime of ABS are approximately 2.0 ns, and it was easy to identify ABS in the mixture. As shown in Figure 12, according to the previously established phasor fingerprint library of four microplastics, the positions of phasor clusters of microplastics and sediments can be visually identified and distinguished in the fluorescence lifetime images.

### 3.7. Validation Results of Raman Spectroscopy

**Figure 13:**
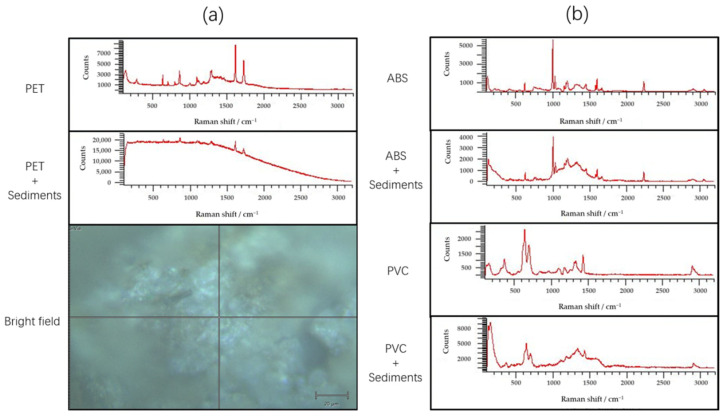
Raman spectra: (**a**) from top to bottom: PET, PET and sediment mixtures, and sediment-covered PET; (**b**) from top to bottom: ABS, ABS and sediment mixtures, PVC, PVC and sediment mixtures.

When detecting microplastics covered with sediment, the baseline of the Raman spectrum is significantly higher due to the presence of unknown matrix interference. For example, when PVC was fully covered by sediment, its characteristic peaks were hardly obvious, which seriously interfered with the detection results, and the PVC components could not be matched after searching the database (Figure 13a). After subtracting baseline and smoothing the Raman spectral data, it could still be seen that the characteristic peaks between the Raman shifts from 1000 to 1500 cm^−1^ were seriously affected by matrix interference (Figure 13b), although there were times when the corresponding microplastic components could be compared, the credibility of the comparison with the database would be greatly reduced.

## 4. Discussion

Despite the increasing development of FTIR and Raman spectroscopy technology [63,64,65], the scope of application for the detection of microplastics is expanding beyond these techniques. However, the supplementation of FLIM technology also has advantages that cannot be underestimated. At present, we believe that the advantage of this technique over the two former techniques is that it can be detected; detection can be achieved quickly without analyzing single plastic samples one by one, and a variety of microplastics in the imaging field can be identified and characterized simultaneously through the selection of phasor clusters. By circling different phasor clusters, different pseudocolors can be added to each microplastic in the fluorescence lifetime image to obtain information on the spatial distribution and abundance of various substances. The detection speed depends on the speed of photon accumulation. The detection time of microplastics with strong autofluorescence, such as ABS, can be as low as a few seconds. The size of the detection range is related to the magnification during imaging. This technique may also be used in biotoxicological experiments to track the distribution and metabolism of microplastics with strong autofluorescence. Because in this technique, fluorescence lifetime detection is carried out on high-resolution confocal microscopy platform [66], the photon numbers of single pixels are accumulated and analyzed, so the spatial resolution is very high, and the method can theoretically detect nanoscale microplastics. The verification experiments showed that microplastics with fluorescence lifetime fingerprints can be effectively identified without complex pretreatment when there are single interfering compounds such as SiO_2_, chitin, toxic compounds, and even complex sediments. This technique is a noninvasive, nondestructive technique using nonionizing radiation. However, the further popularization and application of this technology in the detection of microplastics in the environment depends on the establishment of a fluorescence lifetime fingerprint database, which is a long and tedious task. At this stage, a common microplastic phasor fingerprint library can be established for target experiments—to complete the rapid identification, differentiation, and abundance assessment of environmental samples. Compared with the rapid evaluation method of Nile red staining, this method can greatly improve the accuracy of detection, and can simultaneously characterize the morphology, distribution information, and fluorescence lifetime characteristics of microplastics, reducing misjudgment of nonplastic polymers and overestimation.

Because the fluorescence produced by Nile red dye is stronger than that of microplastics, the time needed to accumulate an effective photon number is faster when detecting Nile red-stained microplastics than unstained microplastics by FLIM, and the detection time can be decreased by at least two times. However, the fluorescence lifetime is also affected by many external factors, including molecular interactions, pH, and temperature [36,67,68]. In this study, ABS particles formed polymers after Nile red staining; such polymer formation may change the polarity of microplastics and transport Nile red to the polymer network of particles due to the influence of solvents, resulting in unstable fluorescence lifetime of microplastic components. Therefore, when measuring the fluorescence lifetime, there will be great differences in the measurement of polymers of different sizes, and the difficulty in distinguishing overlapped phasor clusters is the disadvantage of this technique, which may also exist in the case of unstained microplastics.

In this study, the autofluorescence characteristics of four kinds of microplastics were tested. Although the short wavelength of the ultraviolet band is more effective for plastic excitation, 405 nm is generated by an all-solid-state laser and cannot be used to measure fluorescence lifetime. In the range of WLL, a 440 nm laser can effectively excite most autofluorescence signals. If deep-ultraviolet pulsed light at a wavelength of 200–300 nm can be used, it may be more effective than visible and infrared light [69], which could reduce frame accumulation and shorten the collection time for fluorescence lifetime images. Although there was some emission in the spectra beyond 545 nm, phasor clusters with distinct properties could not be formed from the fluorescence lifetime measurements.

We used the phasor method to analyze FLIM data results, which is suitable for unbiased raw data without any approximation. This method does not require a priori knowledge of the sample to be imaged and produces immediate results. Through this method, we can obtain the attenuation information of each pixel in the image. Even if a single pixel contains multiple types of fluorescent molecules, it can be easily identified. Another advantage of the phasor method is the real-time visualization of the spatial positions of the identified molecular species in the image. The optimized phasor analysis conditions are suitable for the distinction of autofluorescence lifetimes in the phasor diagram and produce results dispersed in the center of the universal circle.

## 5. Conclusions

This study proves that without staining or other processing, and without fitting the original autofluorescence lifetime data measured for microplastics, microplastic particles can be identified and distinguished with FLIM-phasor analysis and a microplastic phasor “fingerprint database”; thus, phasor mapping can be used to identify substances with different fluorescence lifetimes, obtain size and shape information for micron-sized or even nanometer-sized microplastics, and obtain additional information about the spatial distributions and interactions of microplastics with the environment. This method improves the detection efficiency and abundance evaluation accuracy, which provides a new technical direction for studying the distribution and impact of microplastic pollution in the environment and organisms. Considering the complex forms and distribution of microplastic samples in water environments, exploring how to quickly and effectively prepare actual environmental samples and improve the microplastic phasor fingerprint database is the direction of future work.

## Data Availability

The datasets used and/or analyzed during the current study are available from the corresponding author on reasonable request.
